# General Critical Care, Temperature Control, and End-of-Life Decision Making in Patients Resuscitated from Cardiac Arrest

**DOI:** 10.3390/jcm12124118

**Published:** 2023-06-18

**Authors:** Athanasios Chalkias, Georgios Adamos, Spyros D. Mentzelopoulos

**Affiliations:** 1 Department of Anesthesiology, Faculty of Medicine, University of Thessaly, 41500 Larisa, Greece; 2 Outcomes Research Consortium, Cleveland, OH 44195, USA; 3 First Department of Intensive Care Medicine, National and Kapodistrian University of Athens Medical School, 10675 Athens, Greece; george.adamos1983@gmail.com (G.A.); sdmentzelopoulos@yahoo.com (S.D.M.)

**Keywords:** cardiac arrest, post-resuscitation care, intensive care medicine, critical care, outcome

## Abstract

Cardiac arrest affects millions of people per year worldwide. Although advances in cardiopulmonary resuscitation and intensive care have improved outcomes over time, neurologic impairment and multiple organ dysfunction continue to be associated with a high mortality rate. The pathophysiologic mechanisms underlying the post-resuscitation disease are complex, and a coordinated, evidence-based approach to post-resuscitation care has significant potential to improve survival. Critical care management of patients resuscitated from cardiac arrest focuses on the identification and treatment of the underlying cause(s), hemodynamic and respiratory support, organ protection, and active temperature control. This review provides a state-of-the-art appraisal of critical care management of the post-cardiac arrest patient.

## 1. Introduction

For decades, the main focus of resuscitation research was the quality and effectiveness of cardiopulmonary resuscitation (CPR), which led to an increased probability of a return of spontaneous circulation (ROSC). In recent years, optimizing neurologically intact survival from cardiac arrest has been established as the primary aim of resuscitation scientists. Nevertheless, survival rates with good neurological outcomes vary substantially and depend on the location and circumstances of the arrest, the medical team’s ability to restore perfusion to the heart, and the quality of post-resuscitation care [1,2,3].

The unique and complex pathophysiologic mechanisms underlying post-resuscitation disease increase the complexity of management. Patient-related factors, etiology of cardiac arrest, and initial rhythm can further complicate medical care and have a critical impact on outcomes. Unfortunately, the variation in post-cardiac arrest management and the lack of well-organized regional cardiac arrest centers remain important concerns and hamper international efforts toward the standardization of care. 

Despite advances in resuscitation science, neurologic impairment, and multiple organ dysfunction cause considerable mortality and morbidity. High-quality post-resuscitation care has significant potential to reduce early mortality but requires major diagnostic and therapeutic resources and specific training. This review provides a state-of-the-art appraisal of critical care management of the post-cardiac arrest patient.

## 2. Pathophysiology

Post-cardiac arrest syndrome is a complex entity, including myocardial dysfunction, brain injury, the effects of ischemia and reperfusion, and the precipitating pathology or comorbidities as key features. Furthermore, this syndrome can involve injuries caused during the peri-arrest period and several systemic complications that may occur after ROSC, such as acute respiratory distress syndrome, acute renal failure, refractory shock, and disseminated intravascular coagulation [4]. 

Post-resuscitation myocardial dysfunction is observed in up to two-thirds of patients resuscitated from cardiac arrest, even in the absence of prior cardiac disease, and is characterized by the absence of irreversible damage, as well as normal or near-normal coronary flow [5]. Systolic dysfunction of variable severity is commonly identified, but diastolic dysfunction is less frequently reported [6,7,8]. However, in almost all patients, the syndrome is characterized by reduced global contractility, decreased myocardial compliance, and increased microvascular permeability with myocardial edema, all affecting cardiac output. The latter is further impaired due to myocardial injury, metabolic deviation, energy depletion, electrolyte disorders, and the increased formation of reactive oxygen species, which destabilize membrane depolarization and electrical activity [5,6,9]. 

Post-cardiac arrest brain injury involves a complex cascade of molecular mechanisms, most of which remain unknown. After ROSC, several cytokines are upregulated and promote neutrophil infiltration, while the endothelium becomes more dysfunctional and nitric oxide formation decreases. These result in impaired vasodilation and platelet/neutrophil accumulation, increasing the cerebral microvessel vascular tone and extending tissue injury [5]. The enhancement of blood coagulation, which has already begun during CPR, enhances microthrombosis, while the opening of mitochondrial permeability transition pore results in mitochondrial dysfunction, cytochrome c leakage into the cytoplasm, and activation of delayed neuronal death pathways [10,11,12]. Post-cardiac arrest brain injury may be further exacerbated by microcirculatory failure, impaired autoregulation, hypercarbia, hypo- or hyperoxia, pyrexia, hyperglycemia, anemia, and seizures [5,13,14,15]. In most patients, cerebral blood flow is low within 20 min to 12 h after ROSC (hypoperfusion phase), while the disturbed post-ROSC autoregulation may contribute to the development of secondary brain damage [16]. 

Peri-arrest ischemia and reperfusion injury can also aggravate outcomes. Shortly after reperfusion, cellular edema, altered gene expression, activation of inflammation, and increased production of reactive oxygen species result in tissue trauma and endothelial dysfunction [17,18,19,20]. The latter enhances vascular permeability, vasoconstriction, and local inflammation, creating a vicious cycle that ultimately leads to cell death [21,22,23]. 

Among organ systems, gastrointestinal tract dysfunction is common and characterized by loss of barrier integrity and bacterial translocation. The passage of viable bacteria or endotoxins from the gastrointestinal lumen, through the mucosal epithelium, to extra-luminal tissues, such as the mesenteric lymph nodes and other distant organs, can aggravate inflammation and cause secondary infections [24,25,26]. This phenomenon is usually observed after intestinal ischemia-reperfusion injury [27], but the timing and the associated pathophysiological mechanisms have not been elucidated.

Cardiac arrest and CPR induce a marked increase in pro-inflammatory cytokines, which activate pattern recognition receptors (e.g., Toll-like receptors) and inflammasomes, thereby amplifying the inflammatory response in ischemia-reperfusion injury [28]. The enhanced inflammatory response subsequently activates the hypothalamic–pituitary–adrenal axis [5,29,30]. Furthermore, sudden exposure to large quantities of endotoxin entering the bloodstream can contribute to the potentially devastating cascade of the sepsis-like syndrome, with possible concurrent immune suppression or paralysis [31,32,33,34,35]. Whether induced hypothermia after cardiac arrest attenuates the inflammatory response and increases the risk of subsequent infection remains unknown. However, data from mixed surgical–medical intensive care unit (ICU) patients suggest that induced hypothermia does not affect the immune response in patients with cardiac arrest [36]. Additional research is critically needed to elucidate whether all or only a subset of post-arrest patients will benefit from immune modulation and develop strategies to enhance only the beneficial aspects of the immune response following cardiac arrest.

## 3. Immediate Management following Restoration of Cardiac Activity

Conscious patients with stable hemodynamics and good neurological function usually have an uneventful course while undergoing further diagnostic testing. Unconscious or unstable patients must be repeatedly evaluated using the airway, breathing, circulation, disability, and exposure (ABCDE) approach, and their physiology should be optimized on an individual basis. Further diagnostic evaluation, e.g., transfer to the catheterization laboratory, should follow physiological and vital function stabilization. Another key first step is the re-evaluation of the etiology of cardiac arrest. After ROSC, there is usually more time to re-evaluate the patient and the conditions of cardiac arrest and collect more information from emergency medical services, witnesses, and family. Reversible causes should be rapidly investigated to prevent deterioration and relapse of the arrest.

Electrolyte derangements may be among the most common clinical problems encountered during the post-resuscitation period. Hyper- or hyponatremia may be associated with cellular dehydration and central nervous system damage, while dyskalemias may induce severe arrhythmias. Other abnormalities, such as hypocalcemia, hypomagnesemia, and hypophosphatemia may also be associated with increased adverse events. All physiological derangements should be identified and corrected since they can lead to fatal consequences in the resuscitated cardiac arrest patient (Table 1).

## 4. Airway and Anesthesia Management

Post-cardiac arrest patients have an increased risk of cardiovascular collapse and other complications during the peri-intubation period [42]. These physiological derangements may occur due to pre-existing circulatory failure, chronic diseases, effects of anesthetic agents, and transition to positive pressure ventilation. Although the role of skills in airway management and the presence of a second operator remain important [43,44], expertise in peri-intubation physiological optimization is crucial, and endotracheal intubation should be performed by well-trained physicians [45]. Standard periprocedural monitoring includes peripheral oxygen saturation, waveform capnography, blood pressure, electrocardiogram, heart rate, end-tidal oxygen concentration, and, whenever required, invasive monitoring [46]. 

Peri-intubation desaturation carries a four-fold increase in the adjusted odds of re-arrest, and significant efforts should be made to improve denitrogenation and functional residual capacity and minimize shunting [42,47,48]. High-flow nasal cannula, non-invasive ventilation, and the ‘ramped’ position can be used to optimize the delivery of apneic oxygenation and first-pass success, provided that they do not impair hemodynamics and are tolerated by the patient [49,50,51]. In addition, endotracheal intubation is frequently associated with hemodynamic impairment, especially in post-cardiac arrest patients, and peri-procedural individualized hemodynamic optimization can decrease the risk of cardiovascular collapse [42,52,53]. Peri-intubation optimization of venous return and cardiac contractility in these individuals is crucial. Of note, fluid boluses may be ineffective, and early infusion of norepinephrine with or without an inotrope is usually required [54,55]. In patients with right ventricular failure and/or pulmonary hypertension who cannot tolerate further catecholamine-associated increases in pulmonary vascular resistance, vasopressin could be regarded as the vasopressor agent of choice. 

The choice of sedative and induction agents should be also individualized based on the underlying hemodynamic status and comorbidities [42,52]. Although the merits of direct laryngoscopy vs. videolaryngoscopy for airway management in critically ill patients have been a matter of debate over the last few years, videolaryngoscopy may be of high value for overcoming anatomical difficulties but may also lead to life-threatening complications, including apnea and systemic hypotension. In clinical practice, there are several uncontrollable factors that may deteriorate a patient’s physiology during videolaryngoscopy, such as secretions or blood in the airway, reflux of gastric contents, and failure to recognize profound desaturation/hypotension in the setting of clear laryngeal view [56,57]. 

Maintenance of anesthesia after intubation is usually achieved by a combination of hypnotics, opioids, and neuromuscular blocking agents. Although no definitive evidence exists regarding the best sedation regimen or optimal duration after cardiac arrest, patients must be adequately sedated using agents with short elimination half-life, the lowest possible doses, multimodal monitoring, and daily sedation breaks [58,59]. Midazolam and propofol are the most widely used sedatives, with the former allowing a faster neurological recovery; however, it is associated with a higher need for vasopressor therapy [60,61]. Of note, neuromuscular blocking agents are very useful and can facilitate mechanical ventilation, prevent shivering, and help achieve the target temperature quickly in comatose patients [62]. 

Patients treated with targeted temperature management (TTM) must be deeply sedated. However, TTM is associated with significant pharmacokinetic and pharmacodynamic alterations, which may result in sedative accumulation [63,64,65,66]. Therefore, the dosing of anesthetics may have to be reduced during TTM. The depth of anesthesia should always be monitored using frequent clinical assessment, continuous processed electroencephalogram monitoring and nerve stimulators, and should be carefully adjusted to tailoring drug administration to the individual patient.

## 5. Respiratory Management

The aim of positive pressure ventilation after ROSC is to improve oxygenation whilst minimizing circulatory impairment and other adverse events. Large tidal volumes, high respiratory rate and positive end-expiratory pressure (PEEP), and higher airway pressures may increase ventilator-induced lung injury and worsen hypotension and cerebral blood flow; therefore, they should be avoided, especially in the case of intravascular volume depletion or cardiogenic pulmonary edema [46,67]. Immediately after endotracheal intubation, patients should be mechanically ventilated using a lung-protective strategy with a tidal volume of 6–8 mL kg^−1^, PEEP of ≤5 cmH_2_O, plateau pressure < 30 cmH_2_O, and driving pressure < 14 cmH_2_O. After hemodynamic optimization, PEEP levels can be titrated on an individualized basis, while mechanical power should be kept below 17 J min^−1^, taking into account the driving pressure and respiratory rate [40,41,68]. 

Furthermore, an arterial blood gas sample should be obtained as soon as possible, and the fraction of inspired oxygen should be individualized and titrated upon ROSC to an arterial oxygen saturation of 94% to 96% or even lower in some patients [69]. Specifically, hyperoxia (i.e., arterial partial pressure of oxygen (PaO_2_) > 100 mmHg) and hypoxemia (i.e., PaO_2_ < 65 mmHg) should both be avoided. Although extreme hyperoxia is associated with poor neurologic outcomes [70,71,72,73], “critical” hypoxia does not equate to a specific oxygen concentration. Of note, many tissues function physiologically at levels equivalent to an atmosphere of 5% oxygen, and some at levels as low as 1% oxygen [74,75], and therefore, optimizing perfusion may be more important than setting a specific oxygenation target in anesthetized patients [76,77,78]. 

Whether oxygenation and ventilation targets should be modified in patients treated with TTM remains unknown. However, at a core temperature of 33 °C, the PaO_2_ (and arterial partial pressure of carbon dioxide (PaCO_2_)) determined via an analysis of a warmed sample may be higher than the patient’s actual PaO_2_ (and PaCO_2_); therefore, maintaining an arterial blood gas analysis PaO_2_ between 70 and 100 mmHg may be reasonable in these patients. Furthermore, ventilation may be adjusted to maintain normocapnia (or even mild hypercapnia), especially in anesthetized patients treated with temperature control to 32–34 °C or in patients with decreased metabolism and carbon dioxide production [79]. Indeed, when the core temperature is 33 °C, the patient’s actual PaCO_2_ may be 6 to 7 mmHg lower than the value reported by the blood gas machine [80], while patients with chronic hypercapnia (e.g., chronic obstructive pulmonary disease) may require ventilator adjustments to achieve prehospitalization PaCO_2_ values.

Notably, mild hypercapnia may improve cerebral perfusion and have anticonvulsant, anti-inflammatory, and anti-oxidant effects [81,82]. An observational cohort study of 16,542 patients found a greater likelihood of survival to discharge to home in the hypercapnic group and no difference in in-hospital mortality compared to normocapnic and hypocapnic patients [83]. Another study with 5258 cardiac arrest patients reported that unadjusted hospital mortality was the highest in the hypocapnic group (58.4%), compared with the hypercapnic (56.8%) and normocapnic (49.3%) group (*p* < 0.001). Analyses adjusted for age, lowest glucose, and PaO_2_ revealed that hypocapnia (but not hypercapnia) was significantly associated with in-hospital mortality (*p* < 0.001) [84]. A post hoc analysis of the Japanese Association for Acute Medicine out-of-hospital cardiac arrest (OHCA) registry reported that severe hypocapnia, mild hypocapnia, severe hypercapnia, and exposure to both hypocapnia and hypercapnia were more likely to have a 1-month poor neurologic status compared with mild hypercapnia (reference: exposure to mild hypercapnia, respective adjusted odds ratios (ORs) [95% CI]: 6.68 [2.16–20.67], 2.56 [1.30–5.04], 2.62 [1.06–6.47], and 5.63 [2.21–14.34]) [85]. The clinical practice recommendations on the management of perioperative cardiac arrest (PERIOPCA) also recommend a lung-protective ventilation strategy (reducing tidal volume, plateau pressure, and driving pressure) and a PaCO_2_ of 40–50 mmHg after perioperative cardiac arrest, especially in individuals with cerebral vasospasm or generalized atherosclerosis [86]. 

## 6. Circulatory Management

**Figure 1:**
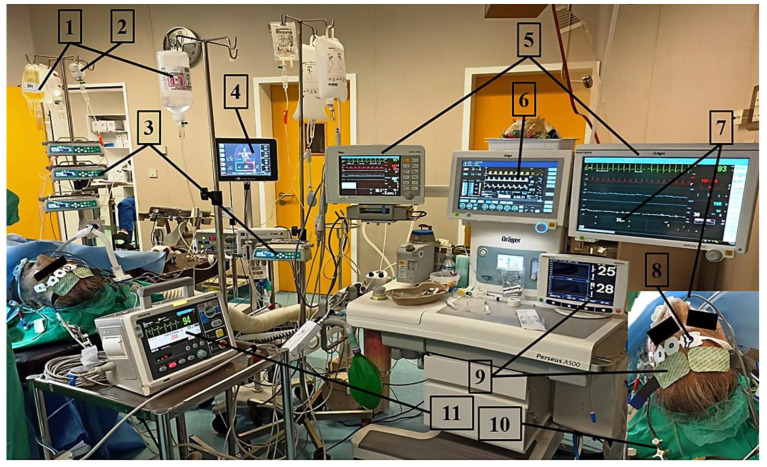
Post-resuscitation care after intraoperative cardiac arrest. A 67-year-old man with a history of end-stage renal disease, heart failure, and pulmonary hypertension underwent emergency surgery (American Society of Anaesthesiologists physical status 5E) due to uncontrolled hemorrhage from an infected axillary-axillary synthetic loop graft. The patient was in hemorrhagic and septic shock upon arrival to the operating room. Two hours after induction of anesthesia, he suffered an intraoperative pulseless electrical activity cardiac arrest. The patient was resuscitated according to the PERSEUS treatment strategy (NCT04428060) [106] and received two i.v. bolus doses of epinephrine 500 μg in order to maintain a diastolic arterial pressure > 40 mmHg during CPR and hydrocortisone 200 mg. A systemic vascular resistance of 1000–1100 dynes s^−1^ cm^−5^, end-tidal carbon dioxide of 15–18 mmHg, and central venous pressure (CVP) of 6–7 mmHg were recorded during CPR. Spontaneous circulation was restored after 2 min of CPR. Post-resuscitation cardiac ultrasound revealed left ventricular hypertrophy with severe systolic dysfunction (LVEF: ~30%, TR: 3^+^/4^+^) and a systolic pulmonary artery pressure of 65–70 mmHg. Inferior vena cava diameter and CVP were 2 cm and 18 mmHg, respectively. The patient was initially supported with noradrenaline 1 μg kg^−1^ min^−1^ and dobutamine 4.17 μg kg^−1^ min^−1^. Depth of anesthesia was adjusted to maintain bispectral index between 39 and 44 with full neuromuscular blockade. Lung-protective ventilation and targeted temperature management (35.2–35.5 °C) were applied. Esmolol infusion was started due to increased heart rate (sinus rhythm 138 beats min^−1^; noradrenaline 1 μg kg^−1^ min^−1^, dobutamine 4.17 μg kg^−1^ min^−1^, esmolol 14.58 μg kg^−1^ min^−1^). Subsequently, arginine vasopressin (AVP) was added to facilitate decatecholaminisation and mitigate the effects of noradrenaline on pulmonary vasculature (noradrenaline 0.4 μg kg^−1^ min^−1^, AVP 0.05 IU min^−1^, dobutamine 2.92 μg kg^−1^ min^−1^, esmolol 14 μg kg^−1^ min^−1^). Thereafter, esmolol was replaced by landiolol in an effort to lower the ventricular rate without markedly deteriorating hemodynamics (noradrenaline 0.37 μg kg^−1^ min^−1^, AVP 0.05 IU min^−1^, dobutamine 2.7 μg kg^−1^ min^−1^, landiolol 6 μg kg^−1^ min^−1^). Tranexamic acid was administrated, and the patient was transfused with a packed red blood cell/fresh frozen plasma/platelets ratio of 2:1:1 (total 6:3:3). Intraoperative time (skin-to-skin) was four hours. After another two-hour intensive care in the operating room, the patient was transferred to the intensive care unit, from which he was discharged 7 days later with a cerebral performance category score of 1. 1: fluid resuscitation and transfusion; 2: total intravenous anesthesia; 3: medical infusion pumps; 4: FloTrac/EV1000 clinical platform; 5: patient monitors providing information in numerical and waveform formats; 6: mechanical ventilator parameters and waveforms; 7: temperature control; 8: bispectral index (BIS) monitoring; 9: regional cerebral oxygen saturation (rSO2); 10: internal jugular vein cannulation; 11: manual external defibrillator/pacemaker.

Myocardial stunning, vasoplegia, and capillary leaks are the main causes of circulatory failure after cardiac arrest and resuscitation, and advanced monitoring may be required in unstable patients to optimize oxygen delivery. Reversible post-cardiac arrest myocardial dysfunction, with a depressed left ventricular ejection fraction and an increased left ventricular end-diastolic pressure, may occur in the hours following ROSC, especially in patients with a longer duration of no-flow or CPR [5]. This impairment may persist for 48–72 h, and early echocardiography can quantify its extent, which may require the use of inotropes [5,87,88,89]. Beta-adrenergic blockade may be necessary as well, as sustained catecholamine-induced β-adrenergic induction produces adverse effects relevant to post-resuscitation management. Indeed, there is evidence suggesting that relative tachycardia is associated with poor neurological outcomes in post-cardiac arrest patients, independently of TTM, and with higher serum lactate levels and admission Sequential Organ Failure Assessment (SOFA) scores [90]. In another study, administration of beta-blocking agents (metoprolol i.v./per os or bisoprolol per os) during the first 72 h of post-resuscitation care was associated with survival at 6 months from the event in both the univariate (*p* < 0.001) and multiple logistic regression analyses (*p* = 0.002) [91]. However, classic β-blockers, such as metoprolol, are not easy to dose in such situations since they may lose their selectivity in the upper standard dose range or when given intravenously, while their longer duration of action may also lead to significant adverse events [92,93]. Ultra-short acting β-blockers, such as esmolol and landiolol, provide significant advantages in these circumstances since their effect can be terminated in a very short time [94,95]. Among these two i.v. agents, landiolol seems to be the most effective for decreasing heart rate in patients with acute heart failure and can be used alongside positive inotropic agents [96,97,98,99,100,101,102,103,104], e.g., in patients with left ventricular dysfunction and increased heart rate. Landiolol is also the only i.v. β-blocker with a specific dose recommendation for these patients [105]. Consequently, landiolol has been used in intensive care patients in conjunction with positive inotropic agents with positive outcomes (Figure 1) [106,107,108,109,110,111,112]. 

Although dobutamine is the first choice for short-term intravenous inotropic support in patients with decreased contractility, the mechanism of action of levosimendan makes it an attractive alternative [113,114]. Levosimendan increases the sensitivity of myocytes to calcium and improves contractility without increasing intracellular calcium levels; the latter is a key pathophysiological mechanism of post-resuscitation myocardial stunning and ischemic contracture, and attenuating this phenomenon seems important [5,115]. Whether post-resuscitation stable dysrhythmias must be treated immediately after their diagnosis remains unknown. However, they are commonly caused by focal cardiac ischemia, and patients with a new-onset dysrhythmia must be evaluated for percutaneous coronary intervention.

In critically ill patients, mean arterial pressure (MAP) represents the entry pressure for the perfusion of most organs and should be maintained >65–70 mmHg [116,117,118]. Although higher MAP levels may be required in patients with brain injury or persistent hypoperfusion (e.g., progressing acute kidney injury or altered mental status) [119,120], adequate circulatory volume, absence of left ventricular outflow tract obstruction, and microcirculatory flow and responsiveness (if possible) should be ideally assessed before using a vasopressor challenge, especially in patients treated with TTM [77,121,122,123]. Considering that organ perfusion pressure is influenced by MAP and venous pressure, maintaining an optimal central venous pressure may facilitate adequate oxygen delivery. Additionally, it is important to remember that diastolic arterial pressure is key for coronary perfusion pressure, and its evaluation is also crucial. Patients with significant or unstable coronary artery disease and those with chronic pulmonary hypertension at a risk of low coronary perfusion pressure may require higher diastolic pressures [124]. 

Vasodilation should be actively treated with vasopressors, initially targeting a MAP of >65–70 mmHg, followed by an individualized approach. Norepinephrine is recommended as a first-choice agent to increase stressed volume and systemic vascular resistance whilst decreasing inflammation-induced capillary permeability [125,126]. Vasopressin can be used as an adjunct to limit the side effects of catecholamines or when agents with a different mechanism of action are needed; however, vasopressin may be the preferred option in patients with pulmonary hypertension, right ventricular failure, and/or vasopressin deficiency [127,128]. Moreover, vasopressin decreases the risk of atrial fibrillation and may improve renal function in patients with vasodilatory shock [129]. Few data are available regarding angiotensin II, limiting its use as a third- or fourth-choice agent in patients with angiotensin II deficiency or altered expression of angiotensin receptors [130,131]. Of note, hypoxic hypercapnia significantly affects the intra- and extrasplanchnic vascular capacitance system, and the dose of exogenous vasopressors should be possibly decreased in these patients to maintain adequate venous return and afterload [132,133,134]. Whether TTM affects hemodynamics remains a topic of discussion as these patients often have diverse requirements in vasopressor support [87,135].

In the context of the vasopressin–steroids–epinephrine (VSE) protocol, early post-ROSC, stress dose steroids may contribute to hemodynamic stabilization, especially in patients requiring high doses of vasopressors (e.g., norepinephrine equivalent ≥ 0.25 μg kg^−1^ min^−1^) and who have multiple organ failure [136,137]. In addition, a re-analysis of combined data from the two randomized VSE trials reported that exposure to stress dose steroids was associated with a lower risk of post-resuscitation lethal septic shock [138]. Nevertheless, a more recent, two-center, randomized trial of stress dose steroids (alone) vs. placebo did not confirm any steroid-associated physiological benefit [139]. 

Optimizing preload during the post-resuscitation period may be difficult, and high doses of balanced crystalloids are often needed. However, avoiding congestion and the injurious effects of fluid over-resuscitation is imperative. An effective fluid resuscitation strategy may necessitate the adoption of a complex, multimodal cardiovascular model capable of primarily integrating both the arterial and venous sides of the circulation, including microcirculatory flow and oxygen extraction [140]. 

Perhaps the most important to recognize is the patient who is fluid responsive but not fluid tolerant because this patient will be harmed by a fluid responsiveness-based strategy [138]. As multiple factors can impact the ability of different organs and compartments to accommodate fluids and maintain their function, and different patient phenotypes exist, frequent multimodal and comprehensive clinical assessments of fluid responsiveness and tolerance are necessary. This assessment may include medical history and physical examination, radiographic evaluation, advanced hemodynamic monitoring, intraabdominal pressure measurement, point of care ultrasound (POCUS), and assessment of abnormalities in splanchnic venous flow patterns (i.e., venous excess ultrasound score—VexUS) [141,142]. 

In patients with refractory circulatory failure, treatment with assistive devices, such as Impella, intra-aortic balloon pump, or veno-arterial extracorporeal membrane oxygenator pumps, may be indicated. However, these devices are often associated with compilations and should therefore be used in selected individuals [143,144,145,146].

## 7. Antibiotic Therapy

Over one-third of adults with OHCA may be bacteremic upon presentation to the Emergency Department [147]. In addition, patients undergoing TTM may develop insulin resistance [148,149], which may impair tissue perfusion and increase the risk of infection. More specifically, clinical and experimental studies suggest that hyperglycemia induces excessive vasoconstriction, endothelial dysfunction, oxidative stress, and inflammatory response, which contribute to microcirculatory dysfunction [77,150,151,152,153]. However, bacteremia and antibiotic administration during resuscitation has not been associated with key outcomes [154]. Therefore, routine prophylactic antibiotics are not recommended despite the possibly increased risk for the development of pneumonia and other infections after cardiac arrest and should be reserved for those with evidence of infection. If antibiotics are administered, significant efforts must be made to improve tissue perfusion and local flow, and thus antibiotic delivery to the potential source of infection.

## 8. Active Temperature Control

In most patients, the primary neurologic injury occurs during cardiac arrest and may continue after ROSC [13]. However, the complex pathophysiology, diverse population, and lack of standardized protocols are major limitations in optimizing neuroprotection. Consequently, post-resuscitation neurological management requires a coordinated multidisciplinary approach aiming at attenuating the progression of cerebral injury. 

Targeted temperature management has been described as the most effective neuroprotective strategy, and current recommendations suggest that it improves neurologic outcomes [69,155,156]. However, the recently published “Hypothermia versus Normothermia after Out-of-Hospital Cardiac Arrest” trial, the largest trial to date, found no difference in survival at 6 months or in health-related quality of life between TTM at 33 °C followed by controlled rewarming or targeted normothermia at 36 °C with early treatment of fever (body temperature > 37.7 °C) [157]. Similar results were reported in the CAPITAL CHILL trial, in which patients were randomly assigned to TTM of 31 °C or 34 °C for a period of 24 h [158]. Nevertheless, that study was underpowered to detect a clinically important difference of ≤3%. 

Although the international guidelines recommend a target core body temperature of 32 to 36 °C and avoiding fever for at least 72 h [69], considerable debate exists on the optimal timing and temperature target, including whether just avoiding fever is enough or whether TTM is also effective for non-shockable rhythms. A target temperature on the higher end of the aforementioned range may be appropriate for patients with mild brain injury, higher bleeding risk, trauma, recent surgery, or septic shock. On the other hand, patients who may benefit from a temperature target of 32–33 °C include those with severe brain injury, subarachnoid hemorrhage, or stroke [159,160,161,162,163,164,165,166]. In addition, the HYPERION trial reported that moderate therapeutic hypothermia at 33 °C for 24 h led to a higher percentage of patients who survived with a favorable neurologic outcome at day 90 compared to targeted normothermia at 37 °C [167]. In a recent randomized trial of nearly 800 patients who received TTM targeting 36 °C for 24 h after resuscitation from cardiac arrest, the composite outcome of death from any cause or hospital discharge at 90 days with either severe neurologic disability or coma was similar for patients who subsequently underwent fever prevention for an additional 12 h (36 h total TTM duration) versus 48 h (72 h total TTM duration) [168] (Table 2).

Based on current evidence and recommendations, the target temperature should be maintained stable for at least 24 h, avoiding variations and shivering, while fever should be avoided for at least 72 h after cardiac arrest [178,184,187,188]. Additionally, patients should be rewarmed at a slow rate (i.e., <0.5 °C h^−1^) [189,190]. 

Interestingly, several recent systematic reviews and meta-analyses of randomized trials do not support the use of TTM. Elbadawi et al. analyzed eight randomized studies with a total of 2927 patients and a weighted follow-up period of 4.9 months and reported that TTM was not associated with improved survival or neurological outcomes compared with normothermia in comatose patients after cardiac arrest [191]. Another systematic review and network meta-analysis of temperature targets found that mild, moderate, or deep hypothermia may not improve survival or functional outcome after OHCA and may be associated with more harm than benefit [192]. Granfeldt et al. assessed all aspects of TTM, including timing, temperature, duration, method of induction and maintenance, and rewarming in 32 trials and reported that the use of TTM at 32–34 °C, when compared to normothermia, did not result in improved outcomes [193]. In another recent systematic review and Bayesian meta-analysis of seven adult cardiac arrest trials, TTM at 32–34 °C for ≥12 h versus normothermia with active control of fever had a chance of ≤53% to ≤78% to reduce the risk of death or unfavorable neurological outcome by 2–4% [194]. Consequently, more high-quality, large, randomized studies are warranted to further clarify the value of targeted hypothermia versus targeted normothermia.

## 9. Prognostication

The overall prognosis of patients following cardiac arrest remains poor, with only half of them surviving to discharge [173,195]. Early prognostication can be difficult, and clinical examination should be initially performed after ROSC and thereafter on a daily basis to assess the neurological status and guide decision making. However, assessments may be confounded by physiological derangements such as hypoxia, hypothermia, circulatory failure, and metabolic acidosis. Most in-hospital deaths in comatose patients are caused by hypoxic-ischemic brain injury [69]. Therefore, the overall prognosis depends on the no-flow time, the quality and duration of CPR, and the quality of post-resuscitation care. Furthermore, the combination of patient characteristics, e.g., age and frailty, components of medical care, anesthesia, TTM, and organ injury mandate that prognosis be determined in most patients only after the first five to seven days after ICU admission [69,196,197,198]. Notably, late awakening may be due to ongoing cardiovascular instability or multiple organ failure and does not preclude full neurological recovery [199,200,201]. 

As accurate prognostication is essential, a multimodal approach should be used in all comatose patients. Brain-computed tomography, measurements of biomarkers such as protein S100B or neuron-specific enolase, evoked potentials, electroencephalogram, and frequent clinical examination are important tools [202]. However, several factors can limit prognostication; for example, TTM may affect the predictive value of computed tomography, while continuous electroencephalogram may have a limited predictive value for a good outcome [203,204,205,206]. Additionally, no clear cut-off has been identified for neuron-specific enolase, and serial sampling at 24, 48, and 72 h after ROSC are necessary to assess trends.

In general, the neurological outcome depends on the prompt restoration of the systemic circulation and adequate oxygen delivery to meet cerebral oxygen demands [207,208]. Until recently, it was assumed that under normal circumstances, autoregulation maintains a constant cerebral blood flow, and changes in mean blood pressure within a range of 50–150 mmHg have a minor influence on cerebral blood flow [209,210]. However, recent evidence suggests that autoregulation maintains cerebral blood flow within a smaller range above baseline MAP [16,211]. After cardiac arrest, the evidence is conflicting, with several studies showing that cerebral autoregulation is preserved after cardiac arrest [212,213], while other studies reported the absence of autoregulation [214]. 

Monitoring cerebral perfusion with transcranial Doppler sonography (TCD) may enhance clinicians’ ability to optimize individual cerebral perfusion, minimize secondary brain damage, and improve prognostication among patients admitted to the ICU after cardiac arrest. This technology allows the measurement of key parameters, e.g., cerebral blood flow velocity and pulsatility index, that allow ongoing, real-time assessments of patients’ autoregulatory indices, intracranial pressure, compliance, and cerebral blood flow, and can identify potentially treatable derangements [215,216]. Various studies have provided conflicting results concerning the association between initial TCD values and neurological outcomes [208,212,217,218,219,220,221].

Although the interpretation of an elevated pulsatility index is complex, values > 1.19 are typically associated with increased downstream cerebrovascular resistance [222]. However, the pulsatility index may increase in the context of decreasing cerebrovascular resistance [217,223]. Transcranial Doppler sonography parameters may complement other available neuromonitoring tools, such as intracranial pressure monitors and near-infrared spectroscopy [224,225]. Of note, TCDs are non-invasive, and their validity may be superior to near-infrared spectroscopy [226]. Real-time data interpretation requires substantial bioinformatic infrastructure and clinician expertise. The GOODYEAR trial (NCT04000334) is anticipated to shed more light on the feasibility of an early goal-directed hemodynamic management with TCD during the first 12 h after ROSC.

## 10. Ethics of Critical Care and End-of-Life Decisions following Cardiac Arrest

Maximizing the benefit of critical care for patients and their families is a key aspect of post-cardiac arrest management. Apart from high-quality organ support, preventing pertinent harm and early end-of-life care decisions are tightly related to the application of patient-centered care. In this context, discussions with the patient or family members following cardiac arrest may affect the quality of care and should rely on a shared decision-making process [227]. The latter can support and optimize the appropriate allocation of resources, decrease ICU/hospital length of stay, aid in the selection of palliative care pathways, and reduce health care costs. 

Effective communication is very important for patient relatives, who may be severely impacted by the illness and critical care stay of their loved ones, experiencing various phycological disorders, such as anxiety, acute stress, post-traumatic stress disorder, and depression [228,229]. Communication in the context of shared decision-making is associated with higher patient/family satisfaction and increased decisional confidence [227,230,231]. However, communication with families is not always easy, and structured communication tools may improve shared decision-making and patient/family satisfaction [232,233]. Consequently, family support interventions that can help reduce these psychological impacts and family-centered communication and care should be key objectives of post-resuscitation management [230,231,232,234,235,236]. Indeed, post-ICU admission-focused discussions with relatives can increase documentation of patient preferences and facilitate advance care planning and end-of-life care [232,237,238,239]. 

Of note, advance care planning is associated with improvements in symptom control and quality of life, decreases in family carers’ decisional conflict, improvements in ICU care and post-resuscitation suffering, lower caregiver burden, and higher patient/family satisfaction [240]. However, an important limitation in the implementation and research in advanced care planning is the lack of a standardized approach; this is also a main cause for the inconsistent findings between studies [238,241,242,243,244,245]. In addition, advanced care planning and shared decision-making may increase organ donation pathways and rates after ensuring family members that donation will be considered only when ongoing treatment cannot improve outcome. 

On the other hand, specific and adequate training of healthcare professionals is imperative for improving critical care and end-of-life decisions following cardiac arrest [246]. However, this type of training is often inadequate during medical training or time of specialization/subspecialization. Easily accessible relevant training programs or workshops to improve the delivery of end-of-life care must be available amongst all hospital staff. For example, the ‘End-of-life Care for All (e-ELCA)’ program is an e-learning library that provides resources to enhance the training and education of the health and social care workforce. The e-ELCA has been highlighted as a resource to help with the implementation of the NICE Guidelines on improving care for people who are in their last days of life [247]. Similarly focused programs created for cardiac arrest patients and their families may enhance the quality of post-resuscitation care.

## 11. Conclusions

A substantial proportion of cardiac arrest deaths can be attributed to the development of post-cardiac arrest syndrome, and post-resuscitation care is the fourth link in the chain of survival. Critical care management requires highly specialized resources and should be based on a multidisciplinary approach ensuring best-practice critical care.

## Figures and Tables

**Table 1:** Common causes of cardiac arrest and their management after admission to the intensive care unit.

Cause	Management
Electrolyte derangements	Urgent correction; medical treatment; continuous renal replacement therapy
Acidosis	Urgent correction; mechanical ventilation; maintain plasma pH > 7.20 and avoid pH normalization (mild acidosis facilitates tissue oxygenation); avoid normal saline (hyperchloremia); use of the anion gap corrected for albumin; initiation of renal replacement therapy when pH < 7.15 in the absence of severe respiratory acidosis and despite other medical treatment interventions
Acute coronary syndrome	Percutaneous coronary intervention; coronary artery bypass graft; optimization of myocardial perfusion; anticoagulation; thrombolysis
Heart failure	Advanced hemodynamic monitoring; deresuscitation/fluid removal; early point-of-care (POCUS) and venous excess ultrasound (VexUS); optimization of intravascular volume; preload, afterload, and heart-lung interactions; medical treatment; mechanical circulatory support; cardiac transplantation
Arrhythmia	Early rate control; correction of electrolyte disorders, acidosis, and other metabolic processes; diagnosis and treatment of abnormal conduction syndromes; medical treatment; cardioversion; pacing
Myocardial trauma	Resuscitative thoracotomy; surgical intervention
Pericardial tamponade	Emergency pericardiocentesis; resuscitative thoracotomy; surgical pericardiectomy or pericardial window
Tension pneumothorax	Emergency decompression; surgical intervention
Pulmonary embolus	Thrombolysis; embolus aspiration; mechanical circulatory support; prevention and treatment of pulmonary hypertension and acute right ventricular failure; surgical intervention
Airway obstruction	Removal of obstacle (e.g., mucus plug); endotracheal intubation; endotracheal/tracheostomy tube exchange; cricothyroidotomy
Asthma/COPD exacerbation	Medical therapies; non-invasive ventilation; high flow oxygen therapy; mechanical ventilation (aiming at improving gas exchange abnormality and avoiding auto-positive end-expiratory pressure); bronchial thermoplasty (within the context of a clinical trial or registry)
Hemorrhage/hypovolemia	Advanced hemodynamic monitoring; fluid resuscitation (patients with maximal vasoconstriction and increased endogenous vasopressin levels may need less fluid); massive transfusion; avoid overload, hemostatic resuscitation, vasopressor use targeted at maintaining perfusion of vital organs *; surgical intervention
Poisoning	Antidote administration; medical treatment; extracorporeal blood purification interventions (e.g., continuous veno-venous hemodiafiltration or hemoadsorption); volume expansion; vasopressor therapy; correction of electrolyte and acid-base disturbances; mechanical circulatory support
Sepsis	30 mL kg^−1^ of crystalloid within 3 h; assess for fluid responsiveness/tolerance; early norepinephrine use; vasopressin when norepinephrine > 0.15 μg^−1^ kg^−1^ min^−1^; use of point-of-care (POCUS) and venous excess ultrasound (VexUS); medical management; source control; early antibiotics; lung-protective ventilation ^#^

* There are more α-1 adrenergic receptors in the veins than in the arteries, and they are abundant in the hepatic veins. In patients with severe hypovolemia, exogenous administration of pure α1-adrenergic receptor agonists will further constrict the already constricted hepatic veins, increasing the impedance of the outflow of blood from the splanchnic system into systemic circulation and leading to sequestration of blood within the liver (preventing auto-transfusion) [37]. In maximally vasoconstricted individuals, large doses of α1-adrenergic receptor agonists would not further increase venoconstriction and will constrict only arteries [37], aggravating the Fåhræus effect (decaying of the relative hematocrit in small vessels as the vessel diameter decreases) and organ perfusion [38,39]. ^#^ Tidal volume should be set between 6 and 8 mL kg^−1^ predicted body weight (volume or pressure controlled); respiratory rate should be initially based on the underlying physiology and thereafter aimed at maintaining normocapnia or mild hypercapnia; plateau pressure should be maintained <30 cmH_2_O and corrected for intra-abdominal pressure when clinically indicated; avoid high positive end-expiratory pressures (set at ≤5 cmH_2_O in patients with ROSC and hemodynamic failure and then individualize); driving pressure should be maintained <14 cmH_2_O; mechanical power should be kept <17 J min^−1^; fraction of inspired oxygen should be titrated to maintain normoxia [40,41].

**Table 2:** Randomized controlled trials on targeted temperature management in adult patients.

Author, Year	Intervention	Inclusion Criteria	Exclusion Criteria	No of Patients	Primary Outcome	Adverse Events	Net Effect of TTM
Bernard et al., 2002 [155].	33 °C vs. 37 °C	Age > 18 y (>50 y for women), OHCA with the initial cardiac rhythm of VF, persistent coma after ROSC	Cardiogenic shock, drug overdose, trauma, cerebrovascular accident, pregnancy	77	Discharge with good neurologic outcome: 49% (hypothermia), 26% (normothermia) (*p* = 0.046); for each 2-year increase in age, 9% decrease in likelihood of good outcome (OR, 0.91; *p* = 0.014); for each 1.5 min in time from collapse to ROSC, 14% decrease in likelihood of good outcome (OR, 0.86; *p* = 0.001); multivariate log regression analysis for good outcome: OR, 5.25 in hypothermia group (*p* = 0.011)	No clinically significant arrythmias in the hypothermia group, no statistically significant differences in platelet and white cell count	Positive
Hachimi-Idrissi et al., 2005 [169].	33 °C vs. 37 °C	Short study period: asystole or in pulseless electrical activity, >18 y, tympanic temperature > 30 °C, GCS < 7; Long study period: age 18–75 y, witnessed cardiac arrest, VF or non-perfusing VT, estimated interval of 5–15 min from collapse to first attempt at CPR, interval of <60 min from collapse to ROSC	Short study period: history of central nervous system depressant drug prior to cardiac arrest, pregnancy, coagulopathy; Long study period: cardiac arrest resulting from intoxication or trauma, responding to verbal command after ROSC, with tympanic temperature <30 °C at admission, evidence of hypotension (mean arterial pressure < 60 mmHg for more than 30 min on admission), terminal illness, pre-existing coagulopathy, pregnancy, unavailability for follow up	28	TTM 33 °C: survival until six months (57%), favorable neurological outcome (43%); Controls: survival until six months controls (43%); favorable neurological outcome (21%)	N/A	Positive
Laurent et al., 2005 [170].	32–33 °C vs. 37 °C	OHCA apparently related to heart disease, age between 18 and 75 years, initial ventricular fibrillation or asystole, estimated interval of <10 min from cardiac arrest to initiation of CPR, and interval of <50 min from initiation of CPR to ROSC	Pregnancy, response to verbal commands after ROSC, or a terminal illness present before the cardiac arrest	42	TTM 33°C: survival until six months (32%), favorable neurological outcome (32%); Controls: survival until six months controls (45%); favorable neurological outcome (45%)	Hypokalemia (45%) Hypophosphatemia (<0.70 mmol L^−1^) occurred in 20 patients (9 in the HF group and 12 in the HF + HT group) and was corrected by intravenous infusion of disodium phosphate, 8 mmol h^−1^, during HF. During the first 24 h in ICU, ventricular tachycardia occurred in 6 patients in the HF + HT group, in 2 patients in HF group, and 3 patients in the control group (*p* = 0.31)	Positive
Hypothermia After Cardiac Arrest Study Group, 2002 [156].	32–34 °C vs. 37–37.5 °C	Age 18–75 y, witnessed cardiac arrest with shockable initial rhythm (VT, VF), arrest of presumed cardiac cause, estimated interval of 5–15 min from collapse to first resuscitation attempt, <60 min from collapse to ROSC	Spontaneous hypothermia < 30 °C, pregnancy, known coagulopathy, terminal disease, comatose state before cardiac arrest, response to verbal commands after ROSC, hypotension for >30 min after ROSC, hypoxemia for >15 min after ROSC	247	Favorable neurologic outcome (CPC 1–2) at 6 mo: 55% (hypothermia), 39% (normothermia) (*p* = 0.009)	Number of patients with any complication: 73% (hypothermia), 70% (normothermia) (*p* = 0.7); sepsis: 13% (hypothermia), 7% (normothermia); lethal or long-lasting arrhythmia: 36% (hypothermia), 32% (normothermia)	Positive
Castrén et al., 2010 [171].	34 °C vs. 35.8 °C	Age > 18 y, OHCA irrespective of initial rhythm, witnessed arrest, CPR initiated within 20 min of collapse	Trauma, drug overdose, cerebrovascular accident, known coagulopathy, asphyxia, or known requirement for supplemental oxygen, electrocution, spontaneous hypothermia, ROSC before randomization, DNR order, transnasal obstruction	194	Median interval from collapse to transnasal cooling: 26 min; median interval from collapse to systemic cooling: 113 min; median time to target core temperature (34 °C): 115 min (transnasal cooling), 284 min (control); mean core temperature on hospital arrival: 35.1 °C (transnasal cooling), 35.8 °C (control) (*p* = 0.01)	Nasal whitening: 14% (resolved in all survivors); epistaxis: 3.2% (serious bleeding in 1 patient with underlying coagulopathy); periorbital emphysema: 1% (resolved within 24 h); total serious adverse events: n = 7 (transnasal cooling), n = 14 (control) (*p* = 0.23)	Neutral
Bernard et al., 2010 [172].	Prehospital TTM 33 °C vs. in-hospital TTM 33 °C	Age > 15 y, OHCA with initial cardiac rhythm of VF, ROSC, systolic blood pressure > 90 mm Hg, cardiac arrest time > 10 min, established IV access	Patient not intubated, dependent on others for activities of daily living before cardiac arrest, spontaneous hypothermia < 34 °C, pregnancy	234	Rate of favorable outcome (discharge home or to rehabilitation facility): 47.5% (early hypothermia), 52.6% (hospital hypothermia) (*p* = 0.43)	No adverse events related to early hypothermia reported	Neutral
Nielsen et al., 2013 [173].	33 °C vs. 36 °C	Age > 18 y, OHCA of presumed cardiac cause irrespective of initial rhythm, >20 min of ROSC	>240 min from ROSC to screening, unwitnessed arrest with asystole as initial rhythm, intracranial hemorrhage or stroke, spontaneous hypothermia < 30 °C	939	All-cause mortality: 50% (33 °C), 48% (36 °C) (*p* = 0.51)	Number of patients with 1 or more complications: 93% (33 °C), 90% (36 °C) (*p* = 0.09); hypokalemia: 19% (33 °C), 13% (36 °C) (*p* = 0.02)	Neutral
Lilja et al., 2014 [174].	33 °C vs. 36 °C	Age > 18 y, OHCA of presumed cardiac cause irrespective of initial rhythm, >20 min of spontaneous circulation after resuscitation	>240 min from ROSC to screening, unwitnessed arrest with asystole as initial rhythm, intracranial hemorrhage or stroke, spontaneous hypothermia < 30 °C	652	Cognitive function assessed by memory, executive function, and attention/mental speed test did not differ between 33 °C and 36 °C group; attention/mental speed was more affected in all patients with cardiac arrest compared with STEMI controls	N/A	Neutral
Kim et al., 2014 [175].	Prehospital TTM <34 °C vs. in-hospital <34 °C	Age > 18 y, ROSC after OHCA irrespective of initial rhythm, endotracheal intubation, established i.v. access, successful placement of esophageal temperature probe, unconsciousness	Trauma, spontaneous hypothermia < 34 °C	1359	Survival to hospital discharge: 62.7% (prehospital hypothermia), 64.3% (control) (*p* = 0.69) (VF); 19.2% (prehospital hypothermia), 16.3% (control) (*p* = 0.30) (non-VF); full recovery or mild neurological impairment at discharge: 57.5% (prehospital hypothermia), 61.9% (control) (*p* = 0.69) (VF); 14.4% (prehospital hypothermia), 13.4% (control) (*p* = 0.30) (non-VF)	Rearrest in the field: 26% (prehospital hypothermia), 21% (control) (*p* = 0.008); use of diuretics within 12 h of hospital admission: 18% (prehospital hypothermia), 13% (control) (*p* = 0.009); PaO_2_ on first arterial blood gas: 189 mm Hg (prehospital hypothermia), 218 mm Hg (control) (*p* < 0.001); evidence of pulmonary edema on first chest x-ray: 41% (prehospital hypothermia), 30% (control) (*p* < 0.001)	Neutral
Debaty et al., 2014 [176].	Intra-arrest TTM 32–34 °C vs. in-hospital TTM 32–34 °C	Age > 18 y, patients with OHCA eligible for advanced life support irrespective of initial rhythm	Trauma, hemorrhage, asphyxia, spontaneous hypothermia < 34 °C, ROSC before randomization, DNR order, pregnancy	245	Median NSE at 24 h: 96.7 μg L^−1^ (intra-arrest hypothermia), 97.6 μg L^−1^ (hospital hypothermia) (*p* = 0.64)	Pulmonary edema: n = 7 (intra-arrest), n = 8 (hospital) (*p* = 0.59); pneumonia: n = 7 (intra-arrest), n = 3 (hospital) (*p* = 0.24); hyperthermia: n = 9 (intra-arrest), n = 5 (hospital) (*p* = 0.36); bacteremia: n = 1 (intra-arrest), n = 0 (hospital) (*p* = 1); hemorrhage: n = 3 (intra-arrest), n = 3 (hospital) (*p* = 0.88); arrhythmia: n = 5 (intra-arrest), n = 7 (hospital) (*p* = 0.39); convulsion: n = 8 (intra-arrest), n = 2 (hospital) (*p* = 0.06)	Neutral
Maynard et al., 2015 [177].	Kim et al. 2014 substudy (Prehospital TTM <34 °C vs. in-hospital <34 °C)	Age > 18 y, ROSC after OHCA irrespective of initial rhythm, endotracheal intubation, established i.v. access, successful placement of esophageal temperature probe, unconsciousness	Trauma, spontaneous hypothermia <34 °C	508	No difference between CPC or mRS scores 3 mo after randomization (*p* = 0.70 and *p* = 0.49, respectively)	N/A	Neutral
Deye et al., 2015 [178].	Advanced endovascular cooling system (34 °C) vs. basic external group (34 °C)	Age 18–79 y, OHCA of presumed cardiac cause, estimated interval of <60 min from collapse to ROSC, <4 h from ROSC to cooling initiation, unconscious patient, availability of endovascular cooling device	Terminal disease, DNR order, pregnancy, uncontrolled bleeding, known coagulopathy, spontaneous hypothermia < 30 °C, OHCA of extracardiac cause, in-hospital cardiac arrest, contraindication to endovascular device, immediate need for ECLS or renal replacement therapy	400	Survival without major neurological damage (CPC 1–2) at day 28: 36.0% (endovascular), 28.4% (external) (*p* = 0.107)	Minor bleeding, hematoma, or Arteriovenous fistula: 43.9% (endovascular), 29.4% (external); microbiological colonization of central venous catheters: 38.5% (endovascular), 26.4% (external); patients experiencing at least 1 cooling-related side effect: 24.6% (endovascular), 14.2% (external) (*p* = 0.0086); 3 patients experienced deep accidental hypothermia (all in external group)	Neutral
Cronberg et al., 2015 [179].	33 °C vs. 36 °C	Age > 18 y, OHCA of presumed cardiac cause irrespective of initial rhythm, >20 min of ROSC	>240 min from ROSC to screening, unwitnessed arrest with asystole as initial rhythm, intracranial hemorrhage or stroke, spontaneous hypothermia < 30 °C	939	Median Mini-Mental State Examination score for all patients, including non-survivors: 14 (33 °C), 17 (36 °C) (*p* = 0.77); median IQCODE score for all patients Including non-survivors: 115 (33 °C), 115 (36 °C) (*p* = 0.57)	N/A	Neutral
Pang et al., 2016 [180].	ECLS TTM 34 °C vs. ECLS TTM 37 °C	Age > 21 y, cardiac arrest irrespective of initial rhythm, interval < 45 min from onset of arrest to ACLS initiation, comatose state, and unresponsiveness after ROSC, intubated with mechanical ventilation, total ACLS time < 60 min	CPR duration > 45 min, severe coagulopathy, drug overdose, head trauma, stroke, pregnancy, terminal illness, spontaneous hypothermia < 30 °C	21	Survival to hospital discharge: 33.3% (hypothermia), 18.2% (normothermia) (*p* = 0.44); survival with good neurologic function (CPC 1–2): 22.2% (hypothermia), 8.3% (normothermia) (*p* = 0.37)	N/A	Neutral
Bernard et al., 2016 [181].	33 °C vs. 36 °C	Age > 18 y, OHCA, established i.v. access, cardiac arrest sustained after initial resuscitation treatment	Trauma, suspected intracranial bleeding, pregnancy, spontaneous hypothermia < 34.5 °C, DNR order	1198	Number of patients transported with ROSC: 33.5% (hypothermia), 39.1% (standard) (*p* = 0.04); overall survival to discharge: 10.2% (hypothermia), 11.4% (standard) (*p* = 0.51)	Acute pulmonary edema: 10.0% (hypothermia), 4.5% (standard) (*p* < 0.001)	Neutral—may cause harm
Scales et al., 2017 [182].	Prehospital cooling at 32–34 °C vs. no prehospital cooling	Age > 18 y, OHCA with sustained ROSC > 5 min, unresponsive to verbal stimuli or requiring intubation	Trauma, burn, spontaneous hypothermia, severe bleeding, severe sepsis, known coagulopathy, DNR order, pregnancy, prisoner status	585	Rate of TT 32 °C–34 °C reached: 30% (prehospital cooling), 25% (standard care) (*p* = 0.22); first temperature measured at hospital admission: 35.1 °C (prehospital cooling), 35.2 °C (standard care) (*p* = 0.53)	Pulmonary edema: 12% (prehospital cooling), 18% (standard care) (*p* = 0.04); use of vasopressors during first 24 h: 54% (prehospital cooling), 62% (standard care) (*p* = 0.04)	Neutral—may increase the application of TTM in hospital
Look et al., 2017 [183].	Internal vs. external cooling	Age 18–80 y, OHCA or IHCA irrespective of initial rhythm, ROSC for >30 min, unresponsiveness after ROSC	Trauma, intracranial hemorrhage, women <50 y, pregnancy, terminal illness, hemodynamic instability	45	Survival to hospital discharge: OR, 3.36 (1.13–10.41) (internal cooling vs. control); OR, 1.96 (0.59–6.86) (internal vs. external); OR, 2.44 (0.95–6.30) (intervention vs. control)	Any cardiac arrhythmias: OR, 0.18 (0.04–0.63) (internal cooling vs. control); OR, 0.26 (0.10–0.70) (intervention vs. control)	Internal cooling method of providing TTM resulted in better survival outcomes compared to controls No significant difference in outcomes for external cooling compared to internal cooling
Kirkegaard et al., 2017 [184].	33 °C for 48 h vs. 33 °C for 24 h	Age 17–80 y, OHCA of presumed cardiac cause irrespective of initial rhythm, sustained ROSC for >20 min, GCS < 8	Unwitnessed cardiac arrest, asystole as initial rhythm	355	Favorable neurologic outcome (CPC 1–2) at 6 mo: 69% (48 h), 64% (24 h) (*p* = 0.33)	Number of patients with 1 or more complications: 97% (48 h), 91% (24 h) (*p* = 0.03); hypotension: 62% (48 h), 49% (24 h) (*p* = 0.013); pneumonia: 49% (48 h), 43% (24 h) (*p* = 0.24); severe bleeding: 4% (48 h), 1% (24 h) (*p* = 0.03)	Neutral
Lopez-de-Sa et al., 2018 [185].	32 °C vs. 33 °C vs. 34 °C	Age 18–80 y, witnessed OHCA of presumed cardiac cause, shockable initial rhythm, interval < 20 min from collapse to CPR initiation, interval < 60 min from collapse to ROSC	Trauma, toxicological cause, pregnancy, DNR order, interval > 240 min from ROSC to randomization, Spontaneous hypothermia < 34 °C, intracranial bleeding, stroke, neurological disability before event, terminal illness	150	Favorable neurologic outcome (mRS ≤ 3) at 90 d: 63.3% (32 °C), 68.2% (33 °C), 65.1% (34 °C) (ns)	Number of patients with 1 or more complications: 84.6% (32 °C), 79.6% (33 °C), 87.8% (34 °C) (ns); respiratory tract infections: 21.2% (32 °C), 49.0% (33 °C), 36.7% (34 °C) (*p* = 0.012)	Neutral
Nordberg et al., 2019 [186].	Prehospital trans-nasal evaporative intra-arrest cooling vs. prehospital standard care Patients admitted to the hospital in both groups received therapeutic hypothermia at 32–34 °C for 24 h	Age 18–80 y, witnessed cardiac arrest irrespective of initial rhythm	Trauma, severe bleeding, drug overdose, cerebrovascular accident, drowning, smoke inhalation, electrocution, hanging, spontaneous hypothermia, anatomical contraindications for nasal catheter, DNR order, terminal illness, pregnancy, known coagulopathy, ROSC before randomization, interval > 15 min from collapse to EMS arrival, need for supplemental oxygen	677	90-day survival with good Neurologic outcome (CPC 1–2): 16.6% (transnasal cooling), 13.5% (control) (*p* = 0.25); 90-d survival with good neurologic outcome (CPC 1–2) in patients with initial shockable rhythm: 34.8% (transnasal cooling), 25.9% (control) (*p* = 0.11); overall 90-day survival rate: 17.8% (transnasal cooling), 15.6% (control) (*p* = 0.44)	Severe cooling-related nose bleeding in 4 patients, pneumocephalus in 1 patient (resolved, patient survived with good neurologic outcome), adverse event rate within 7 d after randomization: 50.4% (transnasal cooling), 48.8% (control)	Neutral
Lascarrou et al., 2019 [167].	32 °C vs. 33 °C Cardiac arrest with non-shockable rhythm	Age >18 y, OHCA or IHCA with non-shockable initial rhythm (asystole or pulseless electrical activity), GCS ≤ 8	Interval > 10 min from collapse to CPR initiation, interval > 60 min from CPR to ROSC, hemodynamic instability, interval of >300 min from cardiac arrest to screening, terminal illness, severe hepatic dysfunction, pregnancy/breast-feeding, lack of insurance	581	Favorable neurologic Outcome (CPC 1–2) at 90 d: 10.2% (hypothermia), 5.7% (normothermia) (*p* = 0.04)	Severe cardiac arrhythmia between 0 and 7 d: 12.3% (hypothermia), 10.4% (normothermia) (*p* = 0.48); seizures between 0 and 7 d: 23.6% (hypothermia), 24.2% (normothermia) (*p* = 0.73); acute pulmonary edema: 6.7% (hypothermia), 8.7% (normothermia) (*p* = 0.33)	Positive
Le May et al., 2021 [158].	31 °C vs. 34 °C	Age ≥ 18 y, OHCA, all cardiac arrest rhythms	Inability to perform activities of daily living, cardiac arrest secondary to intracranial bleeding, severe coagulopathy with clinical evidence of major bleeding, coma not attributable to the cardiac arrest, life expectancy of <1 y due to reasons unrelated to the cardiac arrest	366	All-cause mortality or poor neurologic outcome at 180 d: 89 (48.4%) patients in 31 °C group vs. 83 (45.4%) patients in 34 °C group (risk difference, 3.0% [95% CI, −7.2% to 13.2%]; RR, 1.07 [95% CI, 0.86–1.33]; *p* = 0.56)	Deep vein thrombosis: 21 (11.4%) patients in 31 °C group vs. 20 (10.9%) patients in 34 °C group (risk difference, 0.5% [95% CI, −6.0% to 6.9%]; RR, 1.04 [95% CI, 0.59–1.86]; *p* = 0.88); Thrombus in the inferior vena cava: 7 (3.8%) patients in 31 °C group vs. 14 (7.7%) patients in 34 °C group (risk difference, −3.9% [95% CI, −8.6% to 0.9%]; RR, 0.50 [95% CI, 0.21–1.20]; *p* = 0.11).	Neutral
Dankiewicz et al., 2021 [157].	33 °C vs. 36.5–37.7 °C	Age > 18 y, OHCA of presumed cardiac or unknown cause irrespective of initial rhythm, unconsciousness without response to verbal commands or pain, >20 min of spontaneous circulation after resuscitation	Interval of >180 min from ROSC to screening, unwitnessed cardiac arrest, asystole as initial rhythm, limitations in care, spontaneous hypothermia < 30 °C, ECMO initiation before ROSC, pregnancy, intracranial hemorrhage, severe COPD	1861	6-mo mortality rate: 50% (hypothermia), 48% (normothermia) (*p* = 0.37)	Arrhythmias resulting in Hemodynamic compromise: 24% (hypothermia), 17% (normothermia) (*p* < 0.001); bleeding: 5% (hypothermia), 5% (normothermia) (*p* = 0.81); skin complication: 1% (hypothermia), <1% (normothermia) (*p* = 0.21); pneumonia: 36% (hypothermia), 35% (normothermia) (*p* = 0.75); sepsis: 11% (hypothermia), 9% (normothermia) (*p* = 0.23)	Neutral
Hassager et al., 2023 [168].	Device-based temperature control targeting 36 °C for 24 h followed by targeting of 37 °C for either 12 or 48 h (for total intervention times of 36 and 72 h, respectively) or until the patient regained consciousness	Age ≥ 18 years, OHCA of presumed cardiac cause, Sustained ROSC, unconsciousness (GCS < 8) (patients not able to obey verbal commands) after sustained ROSC	Conscious patients (obeying verbal commands), Females of childbearing potential (unless a negative HCG test can rule out pregnancy within the inclusion window), IHCA, OHCA of presumed non-cardiac cause, e.g., after trauma or dissection/rupture of major artery or cardiac arrest caused by initial hypoxia (i.e., drowning, suffocation, hanging), known bleeding diathesis (medically induced coagulopathy (e.g., warfarin, NOAC, clopidogrel) does not exclude the patient), Suspected or confirmed acute intracranial bleeding, Suspected or confirmed acute stroke, Unwitnessed asystole, known limitations in therapy and Do Not Resuscitate-order, known disease making 180 days survival unlikely, Known pre-arrest CPC 3 or 4, >4 h (240 min) from ROSC to screening, Systolic blood pressure < 80 mm Hg in spite of fluid loading/vasopressor and/or inotropic medication/intra-aortic balloon pump/axial flow device, Temperature on admission < 30 °C	789	Death from any cause or CPC of 3 or 4 at discharge within 90 days (TTM 36 h 32.3%–TTM 72 h 33.6%, *p* = 0.70)	Infection in ICU (TTM 36 h 26.0%–TTM 72 h 27.8%, *p* = 0.56); Arrhythmia in ICU (TTM 36 h 15.8%–TTM 72 h 11.9%, *p* = 0.11); Any bleeding (TTM 36 h 21.4%–TTM 72 h 22.7%, *p* = 0.65); Uncontrolled bleeding (TTM 36 h 4.3%–TTM 72 h 5.3%, *p* = 0.52); Acute kidney injury with renal-replacement therapy (TTM 36 h 9.9%–TTM 72 h 10.6%, *p* = 75; Electrolyte disorder (TTM 36 h 7.6%–TTM 72 h 6.8, *p* = 0.66); Metabolic disorder (TTM 36 h 8.7%–TTM 72 h 7.1%, *p* = 0.41); Seizure (TTM 36 h 21.4%–TTM 72 h 20.2%, *p* = 0.69)	Neutral

OHCA, out-of-hospital cardiac arrest; ROSC, return of spontaneous circulation; GCS, Glasgow coma scale; TTM, targeted temperature management; N/A, not available; VF, ventricular fibrillation; VT, ventricular tachycardia; CPR, cardiopulmonary resuscitation; DNR, do not resuscitate; STEMI, acute ST-elevation myocardial infarction; CPC, cerebral performance category; ECLS, extracorporeal life support; mRS, modified Rankin Scale; ECLS, extracorporeal life support; IQCODE, informant questionnaire of cognitive decline for the elderly; ACLS, advanced cardiac life support; IHCA, in-hospital cardiac arrest; EMS, emergency medical services; ECMO, extracorporeal membrane oxygenation; COPD, chronic obstructive pulmonary disease; HCG, human chorionic gonadotropin; NOAC, novel oral anticoagulants.

## Data Availability

Not applicable.
